# Self-Collection of Saliva Specimens as a Suitable Alternative to Nasopharyngeal Swabs for the Diagnosis of SARS-CoV-2 by RT-qPCR

**DOI:** 10.3390/jcm10020299

**Published:** 2021-01-15

**Authors:** Camino Trobajo-Sanmartín, Marta Adelantado, Ana Navascués, María J. Guembe, Isabel Rodrigo-Rincón, Jesús Castilla, Carmen Ezpeleta

**Affiliations:** 1 Department of Clinical Microbiology, Complejo Hospitalario de Navarra, 31008 Pamplona, Spain; marta.adelantado.lacasa@navarra.es (M.A.); ana.navascues.ortega@navarra.es (A.N.); carmen.ezpeleta.baquedano@navarra.es (C.E.); 2 Instituto de Investigación Sanitaria de Navarra (IdiSNA), 31008 Pamplona, Spain; mi.rodrigo.rincon@navarra.es (I.R.-R.); jcastilc@navarra.es (J.C.); 3 Servicio de Apoyo a la Gestión Clínica y Continuidad Asistencial, Complejo Hospitalario de Navarra, 31008 Pamplona, Spain; mguembes@navarra.es; 4 La Red de Investigación en Servicios de Salud en Enfermedades Crónicas (REDISSEC), 28029 Madrid, Spain; 5 Instituto de Salud Pública de Navarra, 31003 Pamplona, Spain; 6 CIBER Epidemiología y Salud Pública (CIBERESP), 28029 Madrid, Spain

**Keywords:** saliva, nasopharyngeal, SARS-CoV-2, COVID-19, RT-qPCR

## Abstract

A nasopharyngeal swab is a sample used for the diagnosis of SARS-CoV-2 infection. Saliva is a sample easier to obtain and the risk of contagion for the professional is lower. This study aimed to evaluate the utility of saliva for the diagnosis of SARS-CoV-2 infection. This prospective study involved 674 patients with suspected SARS-CoV-2 infection. Paired nasopharyngeal and saliva samples were processed by RT-qPCR. Sensitivity, specificity, and kappa coefficient were used to evaluate the results from both samples. We considered the influence of age, symptoms, chronic conditions, and sample processing with lysis buffer. Of the 674 patients, 636 (94.4%) had valid results from both samples. The virus detection in saliva compared to a nasopharyngeal sample (gold standard) was 51.9% (95% CI: 46.3%–57.4%) and increased to 91.6% (95% CI: 86.7%–96.5%) when the cycle threshold (Ct) was ≤ 30. The specificity of the saliva sample was 99.1% (95% CI: 97.0%–99.8%). The concordance between samples was 75% (κ = 0.50; 95% CI: 0.45–0.56). The Ct values were significantly higher in saliva. In conclusion, saliva sample utility is limited for clinical diagnosis, but could be a useful alternative for the detection of SARS-CoV-2 in massive screening studies, when the availability of trained professionals for sampling or personal protection equipment is limited.

## 1. Introduction

A novel β-coronavirus Severe Acute Respiratory Syndrome Coronavirus 2 (SARS-CoV-2) is the causative agent of the current coronavirus disease 2019 (COVID-19) pandemic that has affected the world since December 2019 [1,2]. The rapid spread of SARS-CoV-2, becoming a public health emergency, has forced the need for new diagnostics methods, vaccines, and therapies [3].

The World Health Organization (WHO) recommends the amplification of viral RNA by reverse transcription quantitative real-time polymerase chain reaction (RT-qPCR) from nasopharyngeal (NP) and oropharyngeal (OP) for the diagnosis and monitoring of infected patients for SARS-CoV-2 [4,5,6]. Nevertheless, the NP and OP specimen collection is considered an invasive procedure that may cause patient discomfort or light bleeding. Furthermore, this sample collection requires close contact between the health professional and the patient, which increases the risk of work-related transmission [7,8].

Several studies have suggested saliva as a suitable sample for the detection of respiratory viruses, including the new, SARS-CoV-2 [7,8,9]. The collection and preservation of saliva samples are points of controversy in the different studies [10,11]. Furthermore, these studies present high variability in the methodology and the sample size.

Saliva collection could be easily performed by the patients themselves without the participation of a health care professional which reduces the risk of transmission and minimizes the use of material protection resources [7,9]. Therefore, this study aims to evaluate the utility of saliva samples for the diagnosis of SARS-CoV-2 infection and identify under what conditions a saliva sample could be useful.

## 2. Experimental Section

### 2.1. Patients and Clinical Specimens

From the 6th to the 12th of August 2020, a prospective study was performed among primary healthcare patients suspected of SARS-CoV-2 infection. Consecutive patients who came to an authorized place to collect samples for detection of SARS-CoV-2 were invited to participate in this study. All patients received an informative document with detailed information about the study objective and voluntary participation description. Those who voluntarily accept to participate received a sterile tube without conservation media and a written protocol for saliva suitable self-collection (Protocol S1). Nasopharyngeal swabs were collected by qualified nurses who had previously been trained. At the same time, the participants collected the saliva samples by themselves following Protocol S1. The nasopharyngeal sample was put in a sterile tube containing a viral transport medium. All samples were stored at room temperature until their transport to the clinical microbiology department of the Navarra Hospital Complex. Saliva samples were sent to the microbiology laboratory and frozen at −80 °C until processing.

Among all the pairs of samples received during the study period, the present study initially included all pairs with positive NP samples (*n* = 337) and 337 randomly selected pairs among those with negative NP samples (a total of 674 pairs of samples) to compare the results from NP and saliva samples.

### 2.2. SARS-CoV-2 Detection by RT-PCR Assay

Nasopharyngeal samples were processed with two different methods, 552 samples were extracted using the STARMag 96 × 4 universal extraction system (Seegene, Seoul, Korea) with the Hamilton Microlab STARlet automation robot (Hamilton Company, Reno, NV, USA) and then RT-qPCR was performed using the Allplex^TM^ 2019-nCoV assay (Seegene, Seoul, Korea) on the CFX96 real-time PCR detection system (Bio-Rad, Hercules, CA, USA) following the manufacturer’s instructions. The remaining 122 samples were analyzed on the Cobas^®^ 6800 platform (Roche Diagnostics GmbH, Mannheim, Germany) following the manufacturer’s instructions. Although we used two different PCR kits (subject to laboratory and commercial stock availability), both methods detect the *E* SARS-CoV-2 gene.

All saliva samples were processed individually using the STARMag 96 × 4 universal extraction system and Allplex^TM^ 2019-nCoV assay (Seegene, Seoul, Korea) for the RT-qPCR, following the manufacturer’s instructions.

Saliva samples were collected in sterile tubes that did not contain conservation media, so we decided to see if buffer lysis’s addition before processing influenced the RT-qPCR results. To evaluate the sample processing controlled over the RT-qPCR results obtained, some saliva samples were diluted on a lysis buffer (1:3) and inactivated for 10 min before RNA extraction.

### 2.3. Statistical Analysis

Sensitivity, specificity, and 95% confidence intervals (CI) were calculated to assess patient diagnostic performance with valid result in saliva samples considering the virus’s detection from an NP sample as the gold standard method. Alternatively, we evaluated saliva sensitivity for detection of infection among all patients, by including invalid results as non-positive samples. The kappa coefficient (κ) was used to estimate the agreement between the saliva RT-qPCR and NP swab RT-qPCR results. Stratified analysis was conducted according to the cycle threshold (Ct). For this purpose, two layers were considered: Ct greater and less than 30 because that cycle number is the one that the literature points out as the most relevant as an infective capacity proxy [12,13].

Sensitivity, specificity, and kappa coefficient were evaluated according to the sex, age (≤14 and >14 years), chronic conditions, Ct for NP samples (≤30 and >30), symptoms (yes/no), and saliva processing with lysis buffer (yes/no).

Means and standard deviations were calculated for quantitative variables and percentages for categorical variables. The associations between categorical variables were studied using a Chi-squared test or Fisher’s exact test when necessary. Exact methods were used to obtain 95% confidence intervals (CI) of proportions. The student’s t-test for paired measures was used to compare Ct values between NP and saliva samples.

### 2.4. Ethical Issues

Samples were obtained with the verbal consent of participants. The Ethical Committee for Clinical Research of Navarra approved the study protocol, PI_2020/96.

## 3. Results

### 3.1. Patients’ Description

Of 674 participants with paired samples tested, the median age was 36 ± 19 years old, 55.5% were women, 103 (15.3%) were children (0–14 years), and 571 (84.7%) were aged >14 years. In terms of clinical presentation, 333 (49.4%) patients presented symptoms compatible with COVID-19 disease, and 341 (50.6%) subjects were asymptomatic. No significant differences were observed by sex, age, and chronic conditions between those participants with positive and negative results for SARS-CoV-2 in the NP sample or between those with Ct ≤ 30 and Ct > 30. However, a significantly higher proportion of people with symptoms was observed in patients with SARS-CoV-2 positive result in NP sample, and those with Ct ≤ 30. Among those NP samples which were tested using Cobas^®^ 6800 of Roche Diagnostics, a higher proportion of positive samples were detected but no differences were observed in Ct values (Table 1).

### 3.2. Comparison of RT-qPCR Results between Saliva and NP Samples

**Figure 1:**
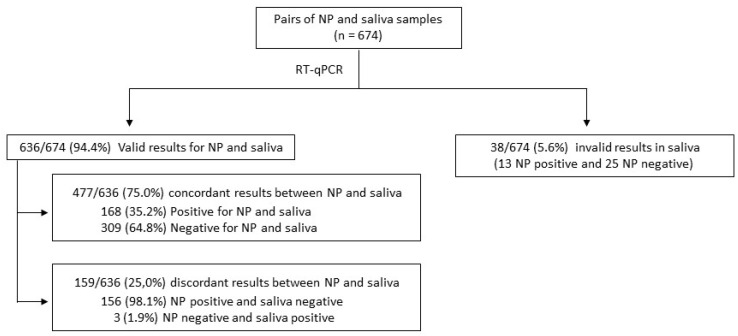
Summary of RT-qPCR results in saliva and nasopharyngeal (NP) samples.

Out of the 674 pairs of samples processed by RT-qPCR, 636 (94.4%) had valid results in both NP and saliva samples. Of the 38 (5.6%) patients with invalid results in saliva specimens, 13 were positive, and 25 were negative for the NP sample. Among the 636 pairs of samples with a valid result, 477 (75.0%) showed concordant results between NP and saliva (168 positives and 309 negatives) and 159 (25.0%) showed discordant results (156 negatives in saliva and positive in NP; and three saliva positive and NP negative) (Figure 1).

When comparing the Ct values for patients with an RT-qPCR positive result in both samples, the median Ct value of NP samples (23.63 ± 6.96) was lower than that of the saliva specimens (28.60 ± 5.94). The difference between means was statistically significant (4.96 ± 5.68; *p* < 0.001).

### 3.3. Sensitivity and Specificity of Saliva Samples

One out of two NP positives was detected with saliva samples. Excluding invalid results, the sensitivity and specificity for saliva samples were 51.9% (95% CI: 46.3%–57.4%) and 99.1% (95% CI: 97.4%–99.8%), respectively. Including invalid results in saliva samples, the sensitivity values were similar, indicating that these data exclusions would not significantly influence the outcome (Table 2). The concordance rate between the two samples was 75% (κ = 0.50; 95% CI: 0.45–0.56) (Table 2).

Virus detection (sensitivity) in samples with Ct ≤ 30 was 91.6% (95% CI: 85.7%–95.6%); however, in the samples with Ct > 30 it was 20.0% (95% CI: 14.4%–26.6%) (*p* < 0.001).

### 3.4. Results under Different Conditions

#### 3.4.1. Age Groups and Chronic Conditions

Among the children, 60% (95% CI: 44.6%–75.4%) of the NP positive patients were also detected in the saliva sample, but sensitivity increased up to 100% (95% CI: 83.3%–100%) in samples with Ct ≤ 30, while only 28.0% (95% CI: 8.4%–47.6%) in those with Ct > 30 (*p* < 0.001). In adults, 50.5% (95% CI: 44.5%–56.6%) of all NP positive patients were also detected in the saliva sample, with a sensibility of 90.2% (95% CI: 84.6%–95.9%), and 19.2% (95% CI: 12.7%–25.7%) in those with Ct ≤ 30 and Ct > 30, respectively (*p* < 0.001). Presence of chronic conditions did not affect the results from saliva samples (Table 2).

#### 3.4.2. COVID-19 Symptoms

In symptomatic patients, saliva samples detected 56.5% (95% CI: 49.1%–63.8%) of NP positive patients, and sensibility was 94.7% (95% CI: 88.1%–98.3%) in those with Ct ≤ 30, but only 16.5% (95% CI: 8.3%–24.7%) of those with Ct > 30 (*p* < 0.001). In asymptomatic patients, saliva samples had a sensitivity of 45.7% (95% CI: 37.0%–54.3%) overall, 85.4% (95% CI: 74.4%–96.4%) in those with Ct ≤ 30, and 24.4% (95% CI: 15.0%–33.9%) in those with Ct > 30. Sensitivity was not statistically significantly different by presence of symptoms (*p* = 0.054) (Table 2).

It should be noted that three cases were detected in NP negative patients with a saliva positive sample. All of them were asymptomatic adult patients with Ct > 35.

#### 3.4.3. Processing with/without Lysis Buffer

Four hundred and seven (60.4%) saliva samples were diluted in lysis buffer, and 267 (39.6%) were processed directly. In the first group, 270 (66.3%) samples showed concordant results between NP and saliva; among those samples processed directly (without lysis buffer), 207 (77.5%) presented concordant results between NP and saliva (*p* = 0.125).

There were no significant differences in sensitivity and specificity by age, COVID-19 symptoms, and the saliva sample processing (Table 2).

## 4. Discussion

Nowadays, a proper diagnosis of COVID-19 is crucial to control the pandemic that is ravaging the world. NP and OP swabs are the recommended specimens to be analyzed by RT-qPCR. However, these samples have several limitations such as the invasive extraction procedure and healthcare professionals’ skills to perform the method. For this reason, it is essential to find a new clinical sample type that is reliable, less invasive, and cost-effective for the detection of SARS-CoV-2 [5,7].

Saliva specimens may be a good alternative for the detection of SARS-CoV-2. The US Food and Drug Administration has approved the use of these samples to diagnose COVID-19 [7,14]. Several studies compared RT-qPCR test results from saliva samples and those from NP or OP samples in patients with suspected or confirmed COVID-19 [5,8,9,15,16]. However, doubts remain regarding the validity of saliva sample results, as the sensitivity may be significantly lower than the NP sample considered as the gold standard.

In the present study, 674 pairs of NP and saliva samples were analyzed by RT-qPCR to validate the utility of saliva samples to diagnose SARS-CoV-2. Considering the NP samples as the gold standard, the saliva samples showed very high specificity (99.1%), which means that the saliva sample is very useful for detecting true negatives since it correctly classifies healthy patients. However, the sensibility was modest (51.9%), so the saliva sample showed a limited capacity to detect true positives. Other studies have also described this fact, with sensitivities for the saliva sample ranging from 31% to 100% [5,8,9,15,17]. However, it is remarkable that our research is the one with the most significant sample size analyzed. Besides, we found a moderate concordance level among NP and saliva samples (κ = 0.50) [16]. This kappa index value obtained in our study is higher than the obtained in a previous study [5], but Pasomsub et al. reported a better kappa value in a study conducted with 200 samples [18]. The variability in results among studies may be due to the sample size, the study’s design, the methodology used, the stage of the patients, and the prevalence of COVID-19 in each region. The present study had higher statistical power than previous studies due to the increased sample size and the inclusion of a similar number of RT-qPCR positive and negative patients. Other advantages were that pairs of samples were obtained simultaneously, and results were evaluated under different conditions.

The RT-qPCR performance on the saliva sample offered high sensitivity for cases with a Ct ≤ 30 and low for those with Ct > 30, regardless of the age, chronic conditions, and presence of symptoms. Despite the discrepancies in the importance of Ct values, a priori the risk of transmission is greater in patients with low Ct. The viral load is high and infectivity could also be higher, particularly for asymptomatic patients or those with mild symptoms [13]. The percentage of false negative results would decrease with repeated samples since high initial cycle times are not durable over time. Although high Ct values can last over time once the disease has passed, there are doubts about these individuals’ infectivity, so the loss of these cases would be less important for epidemiological control and prevention.

Recent studies have demonstrated the utility of saliva samples in asymptomatic patients to detect SARS-CoV-2 [9,17,19]. We analyzed 341 samples from asymptomatic and 333 from symptomatic patients, and no significant differences were observed in the sensitivity, 45.7% and 56.5%, respectively.

We confirmed three cases (1.9% out of confirmed patients) in which the virus was detected in saliva but not in the NP sample. Considering the NP swabs results as the gold standard, and making an exception, these samples could be probably associated with type II error, because of the difficulties in the correct NP swab collection. Other studies have reported similar findings [5,15,17,18,19,20,21].

One of the limitations of this study was the collection of the saliva sample. Although patients were previously informed of the procedure for collecting the saliva samples, if the patient did not follow the instructions, this may affect the results. Different studies suggest that the collection of the sample can influence the result [8,10,20,21,22]. Saliva can be collected directly from the salivary gland canal with buccal or lingual swabs, pouring the saliva that accumulates in the oral cavity into a sterile container or obtaining saliva from the posterior OP [22]. Some authors suggest that the use of saliva from the OP provides higher sensitivity, but if sputum is collected instead of saliva, sensitivity results may be biased, mainly in entirely asymptomatic patients [8,10,22,23].

Regarding the time of collection of the saliva sample, Hung et al. hypothesized that collecting the first hour of the morning could increase the sensitivity [10]. In our case, the saliva samples were collected following the oral cavity accumulation protocol and were not collected from the first hour of the morning, and specimens were frozen at −80 °C until its processing. The present study included NP samples that were tested using two different platforms. However, both methods detect the *E* SARS-CoV-2 gene and are validated for diagnosis. Both platforms are widely accepted as the gold standard for SARS-CoV-2 detection.

Several protocols suggest the use of stabilizing buffers and additives for preserving saliva specimens, and others consider that these buffers are not necessary and may inhibit RT-qPCR [11]. We have evaluated and compared the results of adding or not adding lysis buffer. No statistically significant differences were observed, so the sample treatment with lysis buffer does not seem to influence the results.

## 5. Conclusions

Based on our results, there are several key points to consider for getting definitive conclusions. The procedure of collecting the saliva sample is one of the main points to obtain satisfactory results. Due to its low sensitivity, the saliva sample should not replace the NP sample in all situations. For example, it does not seem suitable for hospital diagnosis, screening in patients who are going to receive health care for other reasons, and high-risk contacts. However, the saliva sample could be a useful alternative to the NP samples in massive screening studies (schools, workplaces, geographical area, etc.) when the availability of trained professionals for sampling or personal protection equipment is limited. Finally, it is essential to highlight that saliva sample collection is a less invasive procedure that can contribute to the patient comfort and may not need health care professional assistance and the subsequent spending of their protective equipment.

## Figures and Tables

**Table 1:** Main characteristics of the patients according to the RT-qPCR result from nasopharyngeal samples.

	Total *n* (%)	Positive *n* (%)	Negative *n* (%)	*p*-Value	Ct ≤ 30 *n* (%)	Ct > 30 *n* (%)	*p*-Value
Sex				0.438			0.786
Female	374 (55.5)	182 (54.0)	192 (57.0)		79 (54.9)	103 (53.4)	
Male	300 (44.5)	155 (46.0)	145 (43.0)		65 (45.1)	90 (46.6)	
Age group				0.705			0.749
0–14 years	103 (15.3)	48 (14.2)	55 (16.3)		20 (13.9)	28 (14.5)	
15–49 years	405 (60.1)	207 (61.4)	198 (58.8)		86 (59.7)	121 (62.7)	
≥50 years	166 (24.6)	82 (24.3)	84 (24.9)		38 (26.4)	44 (22.8)	
Chronic conditions				0.095			0.729
No	499 (74.0)	259 (76.9)	240 (71.2)		32 (22.2)	46 (23.8)	
Yes	175 (26.0)	78 (23.1)	97 (28.8)		112 (77.8)	147 (76.2)	
COVID-19 symptoms				<0.001			0.001
Yes	333 (49.4)	190 (56.4)	143 (42.4)		96 (66.7)	94 (48.7)	
No	341 (50.6)	147 (43.6)	194 (57.6)		48 (33.3)	99 (51.3)	
Lysis buffer processing				0.479			0.119
Yes	407 (60.4)	208 (61.7)	199 (59.1)		82 (56.9)	126 (65.3)	
No	267 (39.6)	129 (38.3)	138 (40.9)		62 (43.1)	67 (34.7)	
RT-qPCR manufacturer				<0.001			0.598
Roche	122 (18.1)	121 (35.9)	1 (0.3)		54 (37.5)	67 (34.7)	
Seegene	552 (81.9)	216 (64.1)	336 (99.7)		90 (62.5)	126 (65.3)	
Total	674 (100)	337 (100)	337 (100)		144 (100)	193 (100)	

*p*-value obtained by Chi-square test of comparison RT-qPCR positive and negative patients.

**Table 2:** Sensitivity (according to Ct and total), specificity, and kappa index of the saliva samples as compared with the result of nasopharyngeal samples.

	Sensitivity, % (95% CI)				Specificity, %	Kappa Index
	All Samples	All Valid Results	Ct ≤ 30	Ct > 30	(95% CI)	(95% CI)
Total	49.9 (44.4–55.3)	51.9 (46.3–57.4)	91.6 (86.7–96.5)	20.4 (14.3–26.6)	99.1 (97.4–99.8)	0.50 (0.45–0.56)
Age group (years)						
Children (0–14)	56.3 (41.2–71.3)	60.0 (44.6–75.4)	100 (83.3–100)	28.0 (8.4–47.6)	100 (93.5–100)	0.62 (0.47–0.76)
Adults (>14)	48.8 (42.9–54.7)	50.5 (44.5–56.6)	90.2 (84.6–95.9)	19.2 (12.7–25.7)	98.9 (96.9–99.8)	0.49 (0.42–0.55)
Chronic conditions						
No	49.8 (43.7–55.9)	51.8 (45.6–58.0)	91.9 (85.6–96.0)	19.6 (13.6–26.8)	100 (93.7–100)	0.50 (0.44–0.57)
Yes	50.0 (39.0–61.0)	52.0 (40.7–63.1)	90.6 (76.6–97.6)	23.3 (12.5–37.6)	96.6 (91.0–99.1)	0.50 (0.38–0.63)
COVID-19 symptoms						
Yes	55.3 (47.9–62.6)	56.5 (49.1–63.8)	94.7 (88.1–98.3)	16.5 (8.3–24.7)	100 (97.5–100)	0.52 (0.44–0.60)
No	42.9 (34.5–51.2)	45.7 (37.0–54.3)	85.4 (74.4–96.4)	24.4 (15.0–33.9)	98.5 (95.5–99.7)	0.47 (0.38–0.56)
Processing						
With lysis buffer	46.2 (39.1–53.2)	49.2 (42.0–56.5)	92.6 (86.3–98.9)	18.4 (10.9–26.0)	99.0 (96.4–99.9)	0.47 (0.39–0.54)
Without lysis buffer	55.8 (46.9–64.8)	55.8 (46.9–64.8)	90.3 (82.2–98.5)	23.9 (12.9–34.8)	99.3 (96.0–100)	0.56 (0.47–0.65)

CI = confidence interval calculated by the exact method.

## Data Availability

All available information is included in this article.

## Supplementary Materials

The following are available online at https://www.mdpi.com/2077-0383/10/2/299/s1. Protocol S1: Saliva sample collection protocol for SARS-CoV-2 RT-qPCR.
