# Supplementary material for: Self-Collection of Saliva Specimens as a Suitable Alternative to Nasopharyngeal Swabs for the Diagnosis of SARS-CoV-2 by RT-qPCR
Source: J Clin Med. 2021 Jan 15;10(2):299. doi: 10.3390/jcm10020299 (PMC7830328; doi:10.3390/jcm10020299)
Supplement: Supplementary file 1 [file jcm-10-00299-s001.pdf]

Supplementary Materials

Protocol S1: Saliva sample collection protocol for SARS-CoV-2 RT-qPCR

In the hour before taking the sample:

- Do not eat or drink;
- Do not discard the saliva you usually produce.

Instructions for obtaining the saliva sample:

1. Collect saliva in your mouth for a few seconds;
2. When you have accumulated saliva, rinse the whole oral cavity with it;
3. Open the green tube you have been given;
4. To expel the saliva, seal the green tube hole with your lips and pour the accumulated saliva until an amount of approximately one finger has been collected. If the amount is less, you should generate more saliva and pour it into the same tube;
5. Close the tube with the cap;
6. Deliver the tube with the saliva sample when requested.
